# Marital rape and its impact on women’s mental health

**DOI:** 10.1192/j.eurpsy.2023.2332

**Published:** 2023-07-19

**Authors:** H. Abrebak, F. Z. Chamsi, A. El Ammouri

**Affiliations:** 1 Faculty of medicine of tangier, Tangier, Morocco

## Abstract

**Introduction:**

In Morocco, the only legal setting for sexuality is marriage, which presumes automatic consent between the couple, as much so that Islam has authorized any sexual practice between spouses except for anal sex .

In our socio-cultural context, it is difficult to determine the extent of the phenomenon, the subject being taboo with a jurisprudence that refuses to recognize the reality of rape between spouses.

**Objectives:**

The objective of this work is to emphasize this phenomenon of marital rape, its psychological repercussions, its legal and religious framework in order to debanalize this phenomenon and act in favor of women’s mental health.

**Methods:**

Our study is conducted on 3 patients seen in liaison psychiatry admitted to the surgical departments for organic complications of medium to severe severity, secondary to rape in the marital context where our expertise was requested in front of the great psychic distress of the latter.

We collected information by taking notes after our psychiatric examination.

**Results:**

Our three patients were victims of rape and violence by their husbands on several occasions, prior to this hospitalization but never reported such an incident. Our patients presented a wide spectrum of psychiatric pathologies: post-traumatic stress disorder, depression, generalized anxiety, suicidal ideas. One of them has acted out on the latter . In addition to these psychological repercussions, two patients had presented complications of a gyneco-obstetrical nature (a miscarriage and a premature delivery). It should be mentioned that these acts of rape in our three patients were always accompanied by physical violence.

**Conclusions:**

In our Moroccan context, rape within a married couple is not yet recognized by our society, which admits that a woman does not have the right to refuse sexual relations to her husband.

Much remains to be done to familiarize and sensitize society to the reality of marital rape and to ensure that women victims obtain adequate support and care.

**Disclosure of Interest:**

None Declared

